# Combination of precipitation and size exclusion chromatography as an effective method for exosome like extracellular vesicle isolation from pericardial fluids

**DOI:** 10.7150/ntno.82939

**Published:** 2023-04-02

**Authors:** Dhananjie Chandrasekera, Rishi Shah, Isabelle van Hout, Willow De Jonge, Richard Bunton, Dominic Parry, Philip Davis, Rajesh Katare

**Affiliations:** 1 Department of Physiology, HeartOtago, School of Biomedical Sciences, University of Otago, Dunedin, New Zealand.; 2 Cardiothoracic Surgery, Dunedin School of Medicine, University of Otago, Dunedin, New Zealand.

**Keywords:** Exosomes, Extracellular vesicles, Biofluids, Precipitation, Size-exclusion chromatography

## Abstract

Extracellular vesicles (EVs), such as exosomes, are nanovesicles that have received significant attention due to their ability to contain various molecular cargos. EVs found in biological fluids have been demonstrated to have therapeutic potential, including as biomarkers. Despite being extensively studied, a significant downfall in EV research is the lack of standardised protocol for its isolation from human biological fluids, where EVs usually exist at low densities. In this study, we tested two well-established EV isolation protocols, precipitation, and size exclusion chromatography (SEC), to determine their efficiency in isolating EVs from the pericardial fluid. Precipitation alone resulted in high yields of low-purity exosomes as tested by DLS analysis, transmission electron microscopy, immunogold labelling and western blotting for the exosomal surface proteins. While EVs isolated by SEC were pure, the concentration was low. Interestingly, the combination of precipitation followed by SEC resulted in high EV yields with good purity. Our results suggest that the combination method can be adapted to isolate EVs from body fluids which have low densities of EV.

## Introduction

Since their discovery over 40 years ago, extracellular vesicles (EVs), in particular, exosomes have been extensively studied for their physiological and pathological roles in the progression of the disease 1-4. Exosomes are nanovesicles with a diameter between 30 and 150 nm. All cells release exosomes into the systemic circulation and body fluids along with the cargo from their host cells 1, 4-7, enabling them to be biomarkers for specific diseases 4, 8-12. In addition, exosomes can affect the host or recipient cells via paracrine, autocrine or even endocrine signalling mechanisms 11, 13-16.

Despite being extensively studied, a significant downfall in EVs research is the lack of standard isolation protocols, particularly for isolating exosomes from human biological fluids 17-20. Initially established EV isolation methods, such as ultracentrifugation, require multiple long centrifugation times at high *g* forces 18. Furthermore, the resulting EVs are still not pure, with large vesicles and proteins 21, 22. Therefore, when considering downstream biomarkers and therapeutic experimental studies, the efficiency of isolating the pure form of EVs is an essential factor in determining their functional and molecular capabilities 19, 22, 23.

Over recent years, multiple products and protocols have been developed to isolate pure EVs for downstream experiments 17-19, 23. Reagent-based protocols use various chemicals that aggregate EVs from biological fluids 19, 23. At the same time, different column-based protocols use membrane-based separation techniques 17, 24. Studies have demonstrated reagent-based precipitation and size exclusion chromatography (SEC) to provide exosome-like EVs suitable for downstream applications 25, 26. However, compared to one another, the two isolation protocols have been demonstrated to yield EVs of varying qualities 17, 27. In the current study, we tested these established protocols to determine their efficiency in isolating exosomes like EVs from the biological fluid. Our results suggest that the combination method could best isolate exosomes like EVs from body fluids with low densities.

## Materials and methods

### Ethics

The Health and Disability Ethics Committee of New Zealand approved the collection of pericardial fluid (PF) which was used as the biological fluid in this study. All the patients provided written consent for collecting and using PF samples in this study.

### PF collection

PF samples were collected from patients (n= 8-10) undergoing either on-pump coronary artery bypass graft (CABG) surgery for ischemic heart disease (IHD), aortic valve repair (AVR) surgery or mitral valve repair surgery (MVR) (Table 1). The PF samples were centrifuged at 250×*g* for 15 minutes at 4°C to remove large tissue debris that would have come through from the sample collection. Additionally, the PF samples were filtered through a 0.2 µm syringe filter to remove any cellular debris. The samples were then frozen and stored at -80°C until used.

### EV isolation

Samples were thawed on ice and aliquoted into three equal portions to isolate the EV using three different isolation protocols. The three protocols tested in this study were precipitation, size exclusion chromatography (SEC) and a combination of precipitation and SEC (now referred to as precipitation + SEC).

#### EV isolation by precipitation

For precipitation, EVs were isolated according to the manufacturer-recommended protocols for Total Exosome Isolation Reagent from other body fluids (Thermo Fischer ™) kit. In brief, the PF sample was mixed with the exosome isolation reagent at a 1:1 ratio and mixed till the suspension was homogenous. The samples were then incubated at 4℃ for 1 hour, followed by centrifugation at 10000×*g* for 1 hour at 4℃. Finally, the supernatant was removed, and the pellet containing EVs was resuspended in 500µl PBS. The samples were stored at -80°C until used.

#### EV isolation by SEC

SEC was performed using qEVOriginal columns (35 nm Legacy column, Izon Science LTD). In brief, 500 µl of PF sample was passed through the temperature-calibrated qEVOriginal column. Once the PF sample had been absorbed through the column, 500 µl of PBS was added to the column, and the flow-through (500 µl, 1^st^ fraction) was collected in a new microcentrifuge tube. This step was repeated nine more times. Fractions 1-6 were discarded as these fractions took up the initial buffer volume sent through the isolation column. While fractions 7-10, which consisted of EVs, were collected and concentrated using Amicon-Ultra 4 centrifugal filters (10 kDa) (Merck Millipore) by centrifuging them at 7000×*g* for 45 min at 4℃. The samples were stored at -80°C until used.

#### EV isolation by the combination of precipitation and SEC

For the combination protocol, EVs were first isolated using the precipitation protocol as above and resuspended in PBS to make a volume of ~500 µl. The resuspended EVs were then passed through the qEVOriginal columns, and the EV fractions were collected as above.

### Characterisation of EVs

The EV samples from the three isolation protocols were characterised for their purity and concentration using following methods.

#### EV particle size measurement

The vesicle density and size were analysed using the Zetasizer Nano ZS DLS instrument (Malvern Instruments, Worcestershire, UK). Before measurement, EV samples were diluted at a 1:20 ratio in PBS to avoid nanoparticle clogging.

A concentration gradient was created to determine the EV concentration using 100 nm calibration particles (Izon Science LTD). The derived particle count rate, in kilo counts per second (kcps), was measured when the concentration gradient of calibration particles was run through the DLS instrument. Similarly, the derived particle count rate of the samples was recorded. Finally, extrapolation from the line of best fit from the calibration curve was used to determine the particle concentration of the EV.

#### Transmission electron microscopy (TEM)

TEM was used to visualize and confirm the presence of exosome-like EVs using the negative staining technique. In brief, 10 µl of the PFA fixed EV solution was applied to the coated side of the plasma-C-copper grid. Once the vesicles were settled onto the grids, the grids were washed with distilled water. The grids were then floated on 50 µL drops of 1% glutaraldehyde, followed by multiple washes with distilled water to wash off excess glutaraldehyde. Next, the grids were transferred to 50 µL drops of 1% uranyl acetate, a contrast agent, for 5 minutes, then transferred to 50 µL drops of a combined methylcellulose (2%) uranyl acetate (4%) on ice for 10 minutes. At the end of the incubation period, the grids were removed using stainless steel loops from the droplets. Excess fluid was removed from the loops by pushing the loop sideways (~60° angle) on a filter paper, leaving behind a thin film of the methylcellulose-uranyl acetate solution. The grids were left to air dry while still on the loops for 5-10 minutes. The stained EVs were imaged using a C100 imager at a magnification range of 66,000 -135,000.

#### Immunogold labelling for CD63

To confirm that the EVs observed under TEM are potentially exosomes, they were labelled with CD63, one of the classical markers of exosomes. For this, 10 µl of the PFA fixed EV suspension was first loaded onto Formvar-carbon-coated Nickel electron microscopy grids and incubated for 20 minutes. The grids were then washed with distilled water and quenched by suspending the grids on 100 µl of 50 mM Glycine in PBS. Following antigen retrieval by incubating with Proteinase K (20 µg/ml) for 10 minutes at 37°C and washing once with dH_2_O, the grids were blocked by being suspended on 100 µl droplets of 1% BSA and 0.1% cold water fish skin gelatin in PBS for 1 hour. Next, the grids were probed with primary antibody against CD63 (1 in 500 dilutions in PBS with 5% BSA, Bio-Rad (Cat # VPA00798), USA) overnight at 4°C. The next day, the grids were washed in PBS, followed by incubation with the Anti-rabbit IgG (whole molecule)-Gold secondary antibody (1 in 15 dilutions in PBS with 1% BSA, Sigma-Aldrich (Cat# G7402), USA) for 1 hour. At the end of the incubation period, the grids were washed five times in PBS, followed by staining with uranyl acetate for 5 minutes to contrast the EVs. The grids were then transferred onto 50 µL drops of methylcellulose (2%)-uranyl acetate (4%) on ice for 10 minutes. After this incubation, the grids were removed from the droplets using stainless steel loops. Excess fluid was removed from the loops by pushing the loop sideways (~60° angle) on a filter paper, leaving behind a thin film of the methylcellulose-uranyl acetate solution. The grids were left to air dry while still on the loops for 5-10 minutes. The stained grids were imaged using a C100 imager at a magnification range of 66,000 -135,000.

#### Western blotting

Western blotting analysis was conducted to determine the expression of classical exosomal markers (CD63, Alix and HSP90). In addition, calnexin and albumin were used as negative markers for exosomes to confirm the absence of any cellular and non-exosomal proteins. Due to low total protein concentrations in the SEC isolation group, the total volumes of the EV samples were directly denatured in a 6× loading buffer. Twenty-five microliters of denatured exosomes from the SEC and the combination isolation groups were loaded into 10% stain-free gels (Bio-Rad™, USA). Due to high concentrations of proteins, exosomes isolated from the precipitation group were diluted five times, and 5 µL was loaded into the stain-free gel.

An additional 5-point protein concentration gradient was added into the gels with an exosome "slurry" of known concentrations ranging from 0.5 µg to 10 µg, consisting of a mix of exosomes isolated by all three isolation protocols 28. This allowed us to determine the gel's exosomal concentration of individual samples. Next, proteins were separated by SDS-PAGE, and the presence of proteins in the gel was confirmed following UV activation. Proteins were then transferred onto the PVDF membrane and probed with antibodies against CD63 (1 in 500 dilutions, Bio-Rad™, Cat # VPA00798, USA), HSP90 (1 in 1000 dilution, Bio-Rad™, Cat # VMA00041, USA), Alix (1 in 1000 dilution, Bio-Rad™, Cat # MCA2493 USA), Calnexin (1 in 1000 dilution, Thermo Fisher, Cat # MA5-32332, USA), and Albumin (1 in 1000 dilution, Bio-Rad™, Cat # VMA00071, USA) overnight at 4°C. The membranes were then probed with secondary antibodies conjugated with horseradish peroxidase (goat anti-mouse (1 in 5000 dilutions, Cat # sc-516102, Santacruz, USA) or goat anti-rabbit (1:3000 dilution, Cat # A6154, from Merck, USA)) at room temperature for 2 hours. Finally, the bands were activated using a chemiluminescence reaction (Clarity Max™ Western ECL Substrate, Bio-Rad™, USA) and visualised in the ChemiDoc™ imaging system (Bio-Rad ™, USA).

### Statistical analysis

GraphPad Prism 8 software was used for the statistical analysis of the results. One-way ANOVA was used to compare the differences between different isolation protocols. P<0.05 was considered statistically significant, and all the data were presented as mean ± the standard error of means (SEM).

## Results

### EVs isolated by precipitation have high yields but low purity

**Figure 1:**
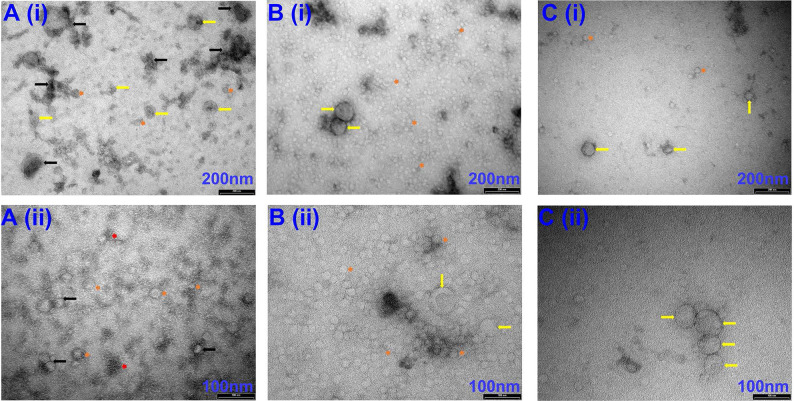
Representative TEM images of EVs isolated via Precipitation (**A**), Precipitation +SEC (**B**) and SEC (C). (i) images captured at 66 k magnification and scale bars are 200 nm. (ii) images at 135 k magnification and scale bars are 100 nm. Yellow arrows indicate individual EVs. Orange short arrows indicate smaller size range exosomes. Black arrows indicate exosome clusters due to leftover aggregation reagents. Red short arrows indicate leftover isolation reagents observed under TEM. N=3 repeated 2 times.

**Figure 2:**
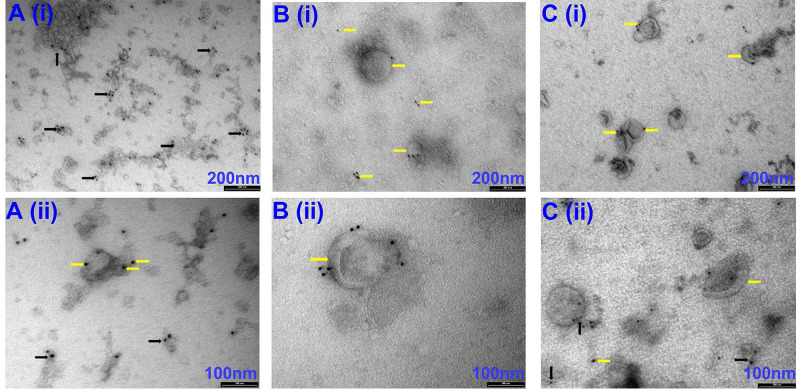
Representative TEM images of CD63-positive EVs isolated via precipitation (A), Precipitation +SEC (B) and SEC (C). (i) images captured at 66 k magnification and scale bars are 200 nm. (ii) images at 135 k magnification and scale bars are 100 nm. Yellow arrows indicate individual EVs labelled with CD63-positive immunogold beads. Black arrows indicate EV clusters labelled with CD63-positive immunogold beads. N=3 repeated two times.

**Figure 3:**
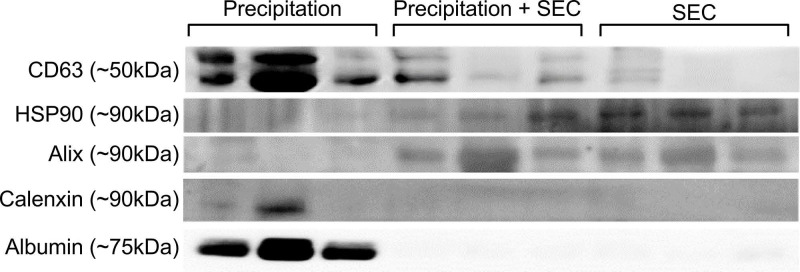
Representative Western Blot images of EVs isolated via precipitation, precipitation + SEC and SEC. The expression of CD63, HSP90 and Alix confirmed the presence of EVs in the samples. Calnexin and albumin were used as a negative control. N=3, all the western blots were repeated at least 2 independent times.

Our initial attempts to image EVs isolated via precipitation were unsuccessful due to the viscous nature of the resulting EV samples (*images not shown*). Therefore, samples were diluted at a ratio of 1:10 with PBS before imaging. Observation under TEM showed exosome-like vesicles aggregated together (**Fig 1A,** black arrows). In addition, remnants of the isolation reagent were observed in the samples, resulting in the formation of vesicle clusters (**Fig 1**, black arrows, **Supplementary Fig 1**, black & red arrows). Furthermore, larger vesicles beyond the EV size range were observed using the precipitation isolation protocol (**Supplementary Fig 1**, green notched arrows). Further, immunogold labelling confirmed the presence of CD63-positive EVs within the clusters of aggregated vesicles (**Fig 2A**), thus confirming the presence of exosomes like EVs in the samples isolated by precipitation. This was further confirmed by western blot analysis, which showed the presence of CD63 (**Fig 3**). Interestingly, EVs isolated by precipitation had no positive expression of other exosomal markers HSP90 and Alix (**Fig 3**). In addition, they also showed the presence of calnexin and albumin, negative markers of exosomes (**Fig 3**) 18.

**Figure 4:**
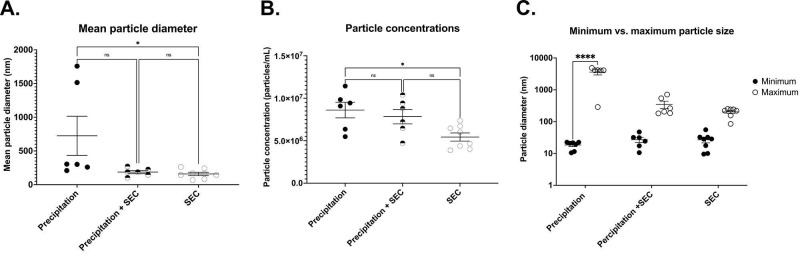
Comparison of EV outputs from different isolation methods. Representative scatter plots for (A) mean particle diameter of EVs, (B) Particle concentration (C) Minimum and maximum particle size. Precipitation n= 5-7, SEC n= 5-7, Precipitation + SEC n= 5-7. Statistical analysis was conducted using one-way ANOVA, p<0.05.

DLS analysis showed the presence of significantly large particles (725.2 ± 290.2 nm n= 6) in the EV samples isolated by precipitation. The average size was remarkably larger than the relative EV size range (**Fig 4A**) 18. This could be because the EVs were aggregated, as observed under TEM (**Fig 1A**). In line with this, the precipitated EVs significantly differed in particle sizes ranging from a minimum of 18.426 ± 1.91 nm to a maximum of 3595.433 ± 673.280 nm (n= 6-7, p= 0.0001 between minimum and maximum particle sizes) (**Fig 4C**), indicating the presence of particles larger than EVs, which could be vesicles aggregated together.

To determine EV particle counts in the samples, we adapted and optimised a protocol from Wallace *et al*. 29 to assess particle concentrations of EV samples, where the concentration of EVs was extrapolated from a known concentration curve. Interestingly, EV isolation by precipitation yielded significantly higher concentrations of exosomes (8.6×10^6^ ± 9.1×10^5^ particles/ml, n= 6, **Fig 4B**), although there was a large variability.

### EVs isolated by SEC have low yields but good purity

TEM imaging demonstrated sparsely spread nanovesicles within the size of EVs (**Fig 1C**). Notably, there were no large-sized vesicles or any aggregated particles. Further, immunogold labelling identified the expression of CD63, thus confirming the vesicles as exosomal EVs (**Fig 2C**). However, due to the sparse presence of EVs, the protein concentration was relatively lower in these samples, which is reflected by the faint expression of exosomal markers CD63, HSP90, and Alix (**Fig 3**). Notably, there was no calnexin or albumin expression in these samples (**Fig 3**), suggesting that the SEC yields pure exosomal like EVs.

Furthermore, DLS particle analysis demonstrated significantly smaller particles in EVs collected by SEC than in precipitation (159.3 ± 290.2 nm, n= 8, p= 0.0047 vs precipitation group) (**Fig 4A**). The average size ranged between a minimum particle size of 26.80 ± 5.279 nm and a maximum of 230.37 ± 34.616 nm, p= 0.9388 between the minimum and maximum particle sizes, **Fig 4C**).

While the above results suggest that SEC isolation yielded pure EVs, one of the caveats was the concentration of EVs. The average concentration was only 5.4×10^6^ ± 4.7×10^5^ particles/ml (n= 8, p= 0.0162 vs precipitation, (**Fig 4B**).

These data suggest that while SEC isolation yields pure EVs within the accepted size range, the yield is significantly lower than the precipitation technique, making it difficult to use for downstream applications.

### EVs isolated by a combination of precipitation and SEC produces pure exosomal like EVs with high yields

Since precipitation resulted in impure EVs with high yields and SEC resulted in pure EVs but low yields, we next tested whether combining both isolation protocols would result in pure EVs with a higher yield. For this, EVs isolated by precipitation were passed through the SEC column to further purify and separate the EVs from any contaminating particles.

TEM imaging confirmed the presence of exosome-like EVs in very high densities without any aggregation (**Fig 1B**). Immunogold labelling confirmed the expression of CD63-positive EVs (**Fig 2B**). Further western blotting analysis showed stable expression of CD63, HSP90 and Alix (**Fig 3**). Furthermore, the absence of calnexin and albumin expression confirmed the absence of contaminating cellular remnants or non-exosomal proteins (**Fig 3**).

Similar to the SEC isolated samples, DLS demonstrated particles sizes that are closer to the exosome size range (187.2 ± 23.97 nm n= 6, p= 0.0696 vs precipitation group, **Fig 4A**) with EV size ranging between a minimum of 27.127 ± 5.232 nm and a maximum of 347.133 ± 91.08 nm, n= 6, p= 0.7803 between the minimum and maximum particle sizes) (**Fig 4C**). Notably, combined isolation resulted in a higher concentration of particles compared to SEC alone (8.4 × 10^7^ ± 7.1 × 10^5^ particles/ml, p= 0.0197 vs SEC, n= 5, **Fig 4B**), which was comparable to the concentration of EVs isolated by precipitation samples (= 0.9896 vs precipitation, n= 6, **Fig 4B**). Altogether, these results indicate that a combination of precipitation and SEC results in high-concentration EVs, which are also pure.

## Discussion

Our results have identified a new combination technique to isolate pure exosomes like EVs with high concentrations from the PF that can be used for downstream experiments. Isolation of pure EVs at optimal concentration is always challenging due to the absence of a standardised protocol. This is more challenging when isolating EVs from body fluids due to low concentration and various cargo in the body fluids 30. In this study, we optimised and compared three protocols to isolate EVs from PF collected from patients undergoing cardiac surgery 7, 31, 32.

Precipitation yielded a high concentration of EVs, which was in line with findings by Helwa *et al*., who used three different commercial kits for isolation by precipitation 23. Due to high yields and guaranteed vesicle stability, Dash *et al*. 33 proposed that precipitation using total exosome isolation kits (Thermo Fisher) is suitable for downstream applications. While both these studies provided an excellent overview of the potential of precipitation-based isolation protocols, there was no report on the purity of the samples. In our study, EVs isolated by precipitation showed the possible contamination with cellular remnants as evidenced by TEM. Furthermore, this study demonstrated that while the precipitation method expressed the classical exosome marker for CD63, which is more abundantly expressed by both exosomes and host cells, they lacked expression of Alix and HSP90 4, 18, 20. Although a five-fold dilution of precipitated exosomes was used for western blot analysis, the larger fraction of non-exosomal contaminants could have interfered with the expression profile of Alix and HSP90, resulting in lower expression of these exosomal marker proteins in the exosomes isolated by precipitation. Furthermore, the presence of non-exosomal contaminants in precipitated exosomes was confirmed by the expression of calnexin, a marker of cellular contamination, and albumin, a common contaminant in EVs isolated from biofluids. 34. Interestingly, Skottvoll *et al*. 35 demonstrated comparable isolation efficiencies between ultracentrifugation and kit-based precipitation techniques. At the same time, both protocols resulted in high yields. Similar to our findings, both methods resulted in the presence of non-exosomal markers 35.

We also observed aggregated clusters of vesicles from the precipitation protocol. This was also evidenced by Dash *et al*. 33 using a kit from the same manufacturer, showing the presence of aggregated vesicles. This is likely due to residual isolation reagents from the precipitation kit and is expected to interfere with the functional efficacy of the EVs. In support of this, Paolini *et al*. 36 demonstrated that the presence of the precipitation reagent in the exosomal samples interfered with exosome biological activity. This was further confirmed by Gámez-Valero *et al*. 37, who demonstrated impaired *in vitro* exosomal function in the presence of a remnant isolation reagent. They also showed reduced cell viability following the introduction of precipitated EVs contaminated with residual isolation reagent 37.

While these limitations can be overcome by isolating EVs using SEC, which was pure without any aggregates or large vesicles, the yield was much lower as evidenced by lower CD63 staining, limiting their availability for downstream experiments. This suggests the need for a higher starting volume of the samples. This is particularly challenging while dealing with clinical samples, which are limited and precious. Other studies have also demonstrated the ability to isolate pure EVs from plasma using SEC. However, like our study, the yield was poor 34, 38.

While multiple studies have individually assessed the efficiency of EV isolation by precipitation and SEC, the potential of a combined protocol on biological fluids has not been evaluated to our knowledge. Seminal evidence showed the possibility of enriching EVs by combining ultracentrifugation and SEC 39-41. Results from this study showed that enrichment of PF EVs by precipitation before purification by SEC yielded pure exosomal like EVs with high concentration, which was also evidenced by TEM and immunogold labelling. The positive expression of exosomal marker proteins CD63, Alix and HSP90 and the absence of contaminant proteins calnexin and albumin in the exosomes isolated by precipitation and SEC further indicated the purity of the exosomes isolated by this protocol. Studies conducted by Martínez-Greene, Hernández-Ortega *et al.* 42 indicated a similar efficiency in EV isolation from cell culture media samples using a precipitation and SEC protocol. Furthermore, their study indicated that the EVs isolated using the combination of precipitation and SEC were readily incorporated into recipient cells 42.

Interestingly, variability in particle concentration was observed across all EV isolation methods. Emanueli *et al.* 10 demonstrated an increased release of exosomes into the circulation in response to CABG surgery. Our study's observed variabilities in EV concentrations could be attributed to the EVs being isolated from PF that were collected from patients undergoing CABG surgery. However, care was taken to have an equal distribution of samples collected from the three surgery types in this study.

In conclusion, considering EV isolation methods is crucial, as this may potentially affect downstream experimentation. Our optimised method of combining precipitation and SEC can be adapted for isolation from body fluids with low EV concentration to isolate pure EVs with high yields suitable for downstream applications. Future studies should focus on determining if the combined isolation protocol enhances the functional ability of the isolated EVs.

## Supplementary Material

Supplementary figures and tables.

## Figures and Tables

**Table 1:** Patient characteristics of samples used for this study. The total number of samples used n= 19.

Parameter	
**Male**	**57.9 %**
**Female**	**42.1 %**
**Age (Years)**	**64 ± 2.4**
**Ejection fraction**	**52.7 ± 2.2**
**Hypertension**	**68.4 %**
**Ex-smokers**	**36.8 %**
**Surgery type**	
**CABG**	**36.8 %**
**AVR**	**31.5 %**
**MVR**	**36.8 %**

## Abbreviations

AVR: aortic valve repair surgery
CABG: coronary artery bypass graft surgery
DLS: dynamic light scatter
EV: extracellular vesicles
HSP90: heat shock protein 90
MVR: mitral valve repair surgery
PBS: phosphate buffered saline
PF: pericardial fluid
SEC: size exclusion chromatography
SEM: standard error of means
TEM: transmission electron microscopy
