# Supplementary material for: Combination of precipitation and size exclusion chromatography as an effective method for exosome like extracellular vesicle isolation from pericardial fluids
Source: Nanotheranostics. 2023 Apr 2;7(4):345–52. doi: 10.7150/ntno.82939 (PMC10161387; doi:10.7150/ntno.82939)
Supplement: Supplementary file 1 — Supplementary figures and tables. [file ntnov07p0345s1.pdf]

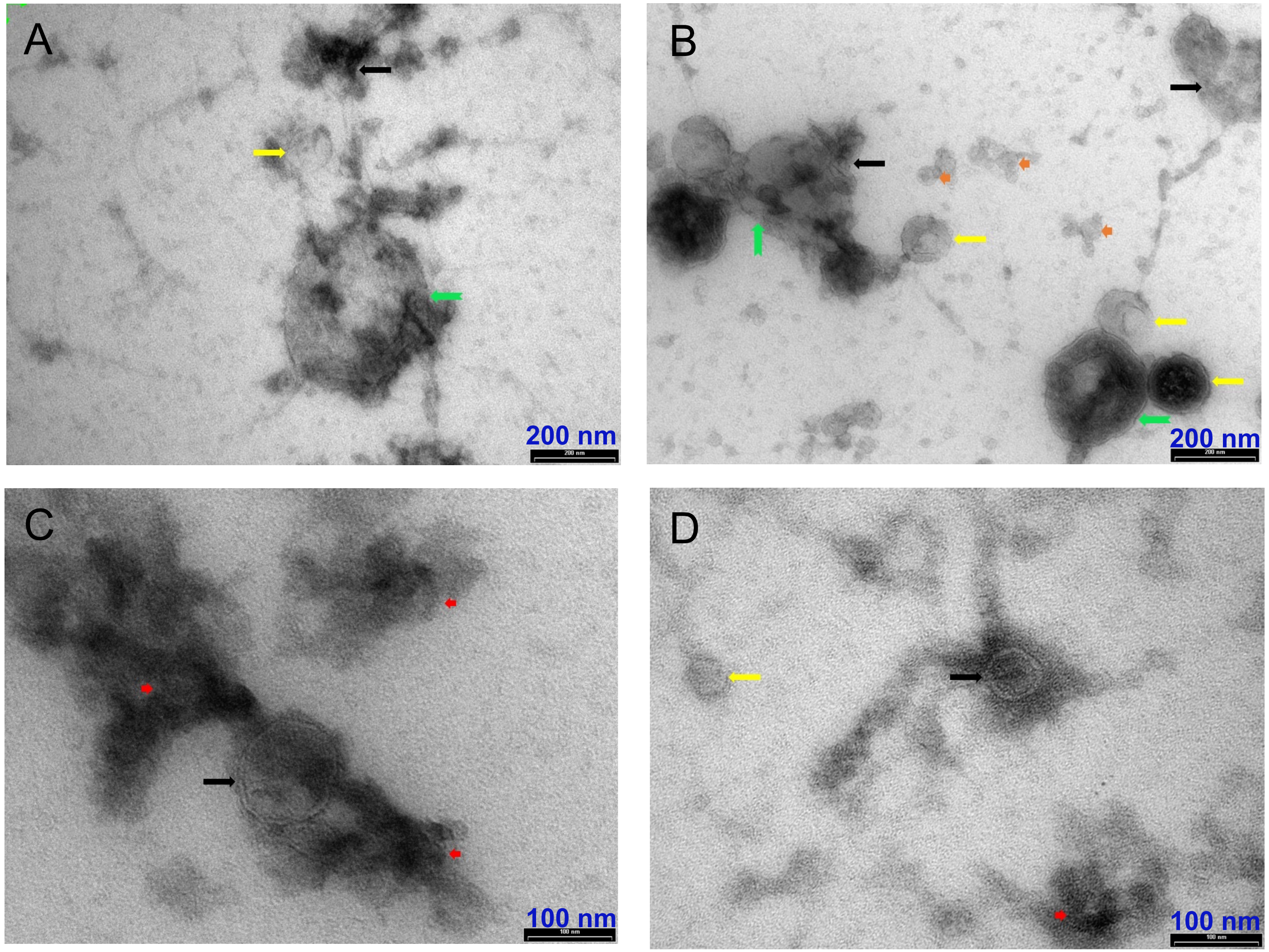

Supplemental figure 1: Representative TEM images of EVs isolated via Precipitation isolation protocol. Images A & B in 66k magnification, with 200 nm scale bar. Images C & D in 135k magnification, with 100 nm scale bar. Yellow arrows indicate mid to large-size range exosomes. Orange short arrows indicate smaller size range exosomes. Green notched arrows indicate large vesicles beyond the exosome size range. Black arrows exosome clusters due to leftover aggregation reagents. Red short arrows indicate leftover isolation reagents observed under TEM. N=3 repeated two times.

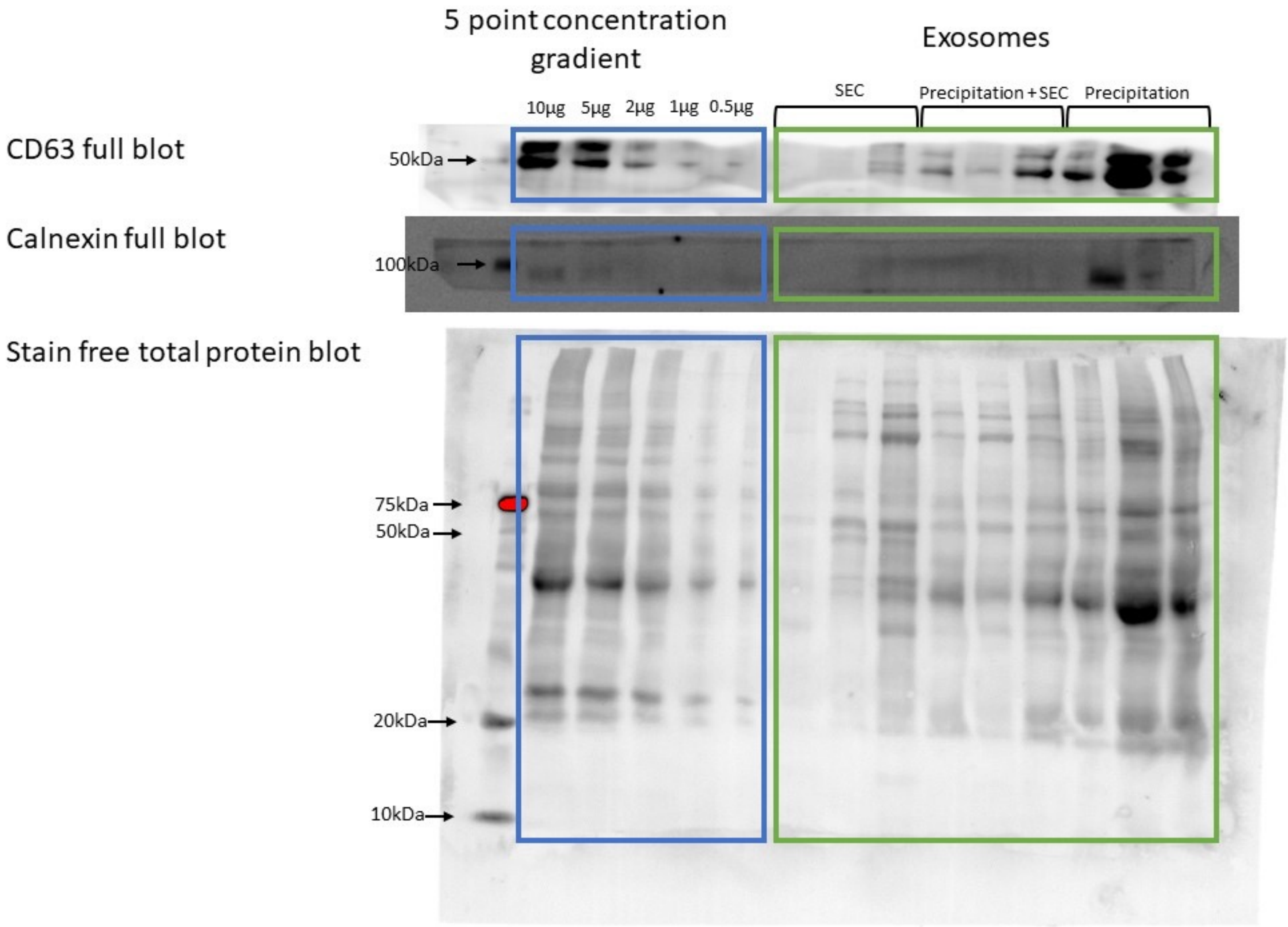

Supplemental figure 2: Full blot images of representative Western Blot for CD63 and Calnexin and stain-free total protein blot. Blue boxes indicate the lanes with the 5-point concentration gradient. Green boxes indicate lanes with EVs. N=3, repeated at least two independent times.

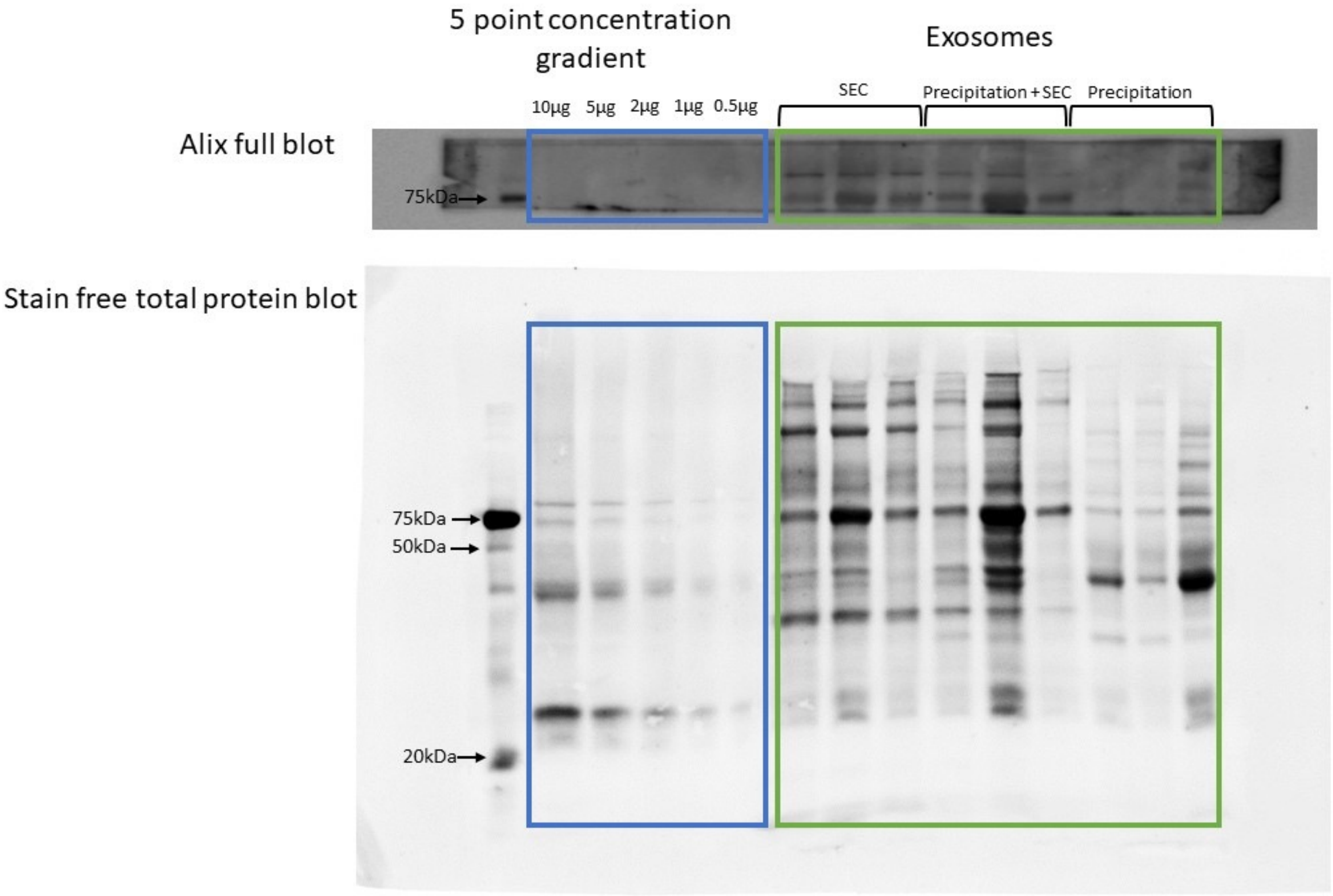

Supplemental figure 3: Full blot images of representative Western Blot for Alix and stain-free total protein blot. Blue boxes indicate the lanes with the 5-point concentration gradient. Green boxes indicate lanes with EVs. N=3, repeated at least two independent times.

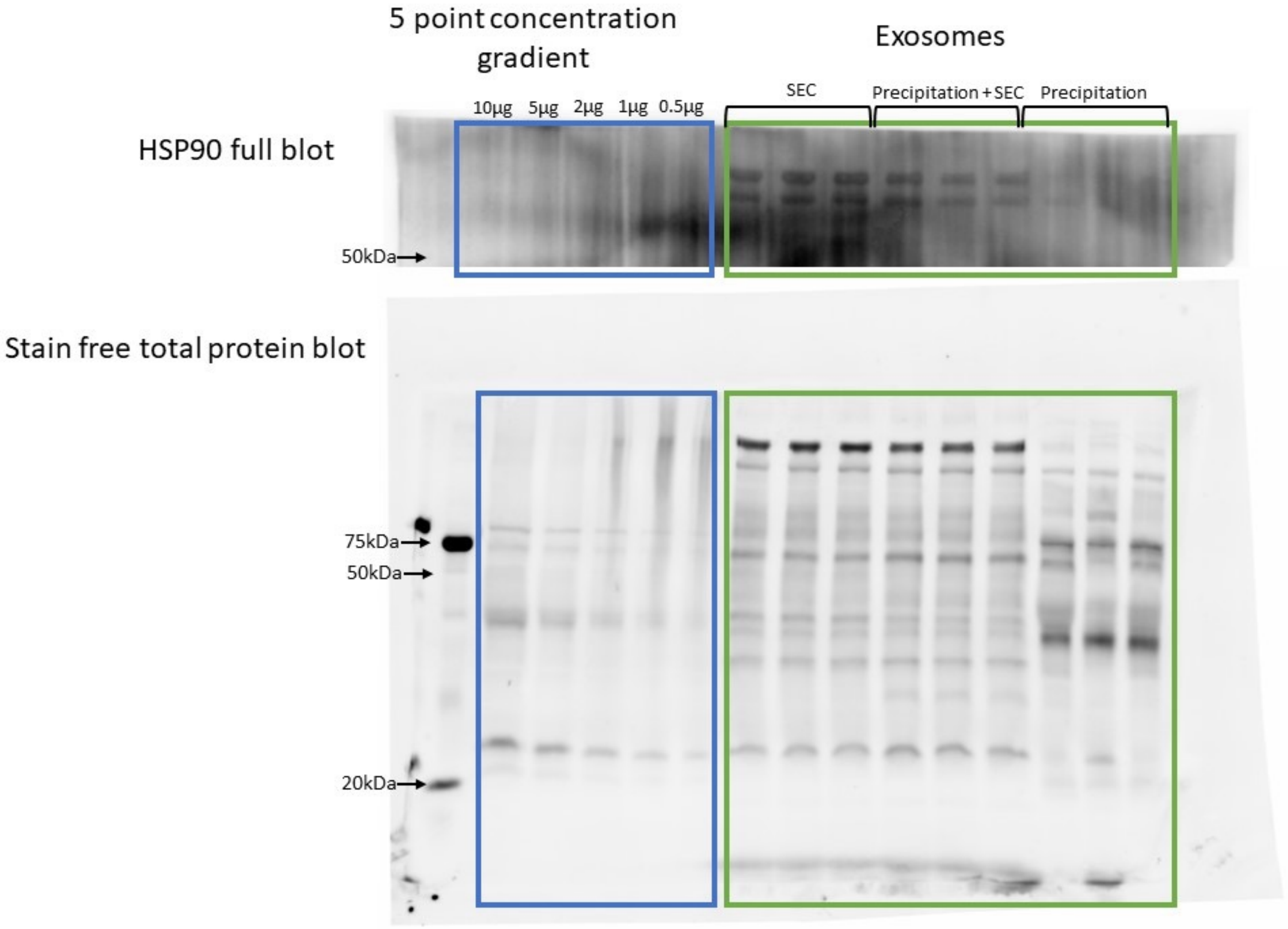

Supplemental figure 4: Full blot images of representative Western Blot for HSP90 and stain-free total protein blot. Blue boxes indicate the lanes with the 5-point concentration gradient. Green boxes indicate lanes with EVs. N=3, repeated at least two independent times.

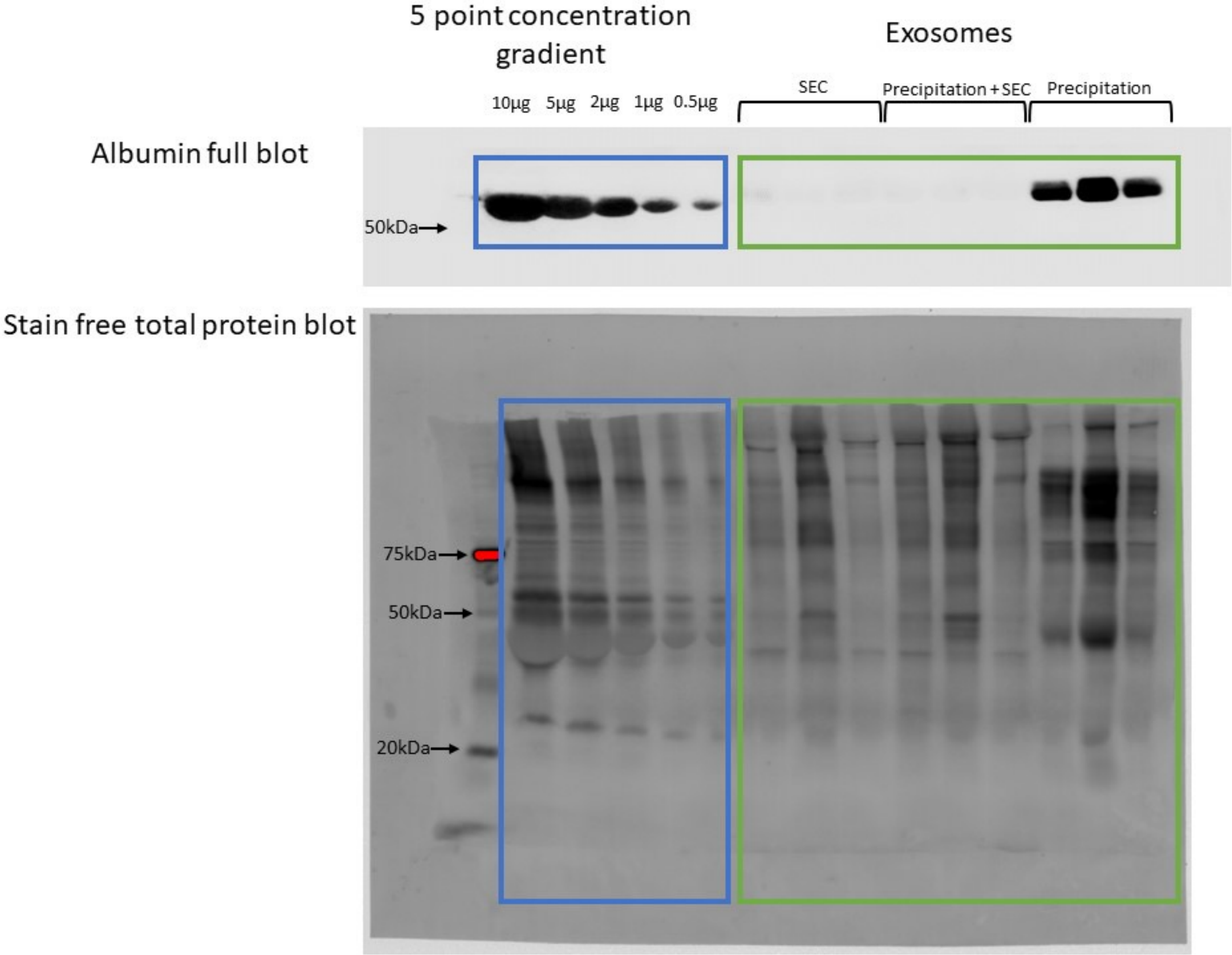

Supplemental figure 5: Full blot images of representative Western Blot for albumin and stain-free total protein blot. Blue boxes indicate the lanes with the 5-point concentration gradient. Green boxes indicate lanes with EVs. N=3, repeated at least two independent times.
